# The Story Retold: The Kocher Manoeuvre

**DOI:** 10.7759/cureus.29409

**Published:** 2022-09-21

**Authors:** Anastasia Livani, Stavros Angelis, Panagiotis N Skandalakis, Dimitrios Filippou

**Affiliations:** 1 Department of Surgery, Agia Sophia Children’s Hospital, Athens, GRC; 2 Department of Anatomy and Surgical Anatomy, Medical School, National and Kapodistrian University of Athens, Athens, GRC

**Keywords:** mesopancreas, fascia of treitz, kocher manoeuvre, pancreas, duodenum

## Abstract

The Kocher manoeuvre is used for mobilization of the duodenum and head of the pancreas and bears the name of Theodor Kocher, who published it in 1903. We describe the embryology of the duodenum and pancreas, relating it to surgical anatomy applied during the procedure. Finally, we present the key points of the procedure, providing more insights into the anatomical structures that are mainly involved.

## Introduction and background

The story

Mobilization of the duodenum was first described by Jourdain in 1895. Theodor Kocher, after whom the Kocher manoeuver was named, in his original work, "Mobilisierung des Duodenum und Gastroduodenostomie" (Zbl. Chir., 1903), provides a detailed description of the procedure: "The loop of the duodenum is freely suspended in the abdominal cavity during embryonic development (Merkel); among children, it is even more movable, but later it lies on its right side on the posterior abdominal wall, adheres or fuses with it, and only its anterior surface is covered by the parietal peritoneum of the posterior abdominal wall. One can confirm this in a cadaver. However, with relative ease, the loop can be so loosened that the pars descendens and the flexor duodeni inferior are as movable as in the embryo and can be displaced anteriorly" [1].

From the above, it is obvious that Kocher knew that the duodenum and pancreas are secondarily retroperitoneal organs that once were freely suspended in the peritoneal cavity by a mesentery. In fact, the Kocher manoeuvre restores the original peritoneal position of these organs and their primordial mesenteries. Since Kocher’s time, a lot more has been discovered about the embryogenesis of the duodenum and pancreas and how they find their final position in the retroperitoneal space. In this study, we will describe those events briefly, giving an outline of the surgical anatomy of the region. Finally, a short discussion about relevant clinical issues, from the scope of embryology and anatomy, will be provided.

## Review

Why? - Events during embryonic and foetal periods

During the third and fourth weeks of gestation at the ventral junction of the foregut and midgut, the primitive duodenum makes its appearance, and by the end of the fourth week, its distinct cell populations have already been differentiated. At about the 24th day of gestation, from the floor of the early duodenum arises the hepatic diverticulum, growing into the septum transversum. From the dorsal side of the duodenum, contained in the mesogastrium, arises the dorsal pancreatic primordium at the end of the fourth week [2]. In the meanwhile, the ventral diverticulum has differentiated into primitive liver cords, the gallbladder and cystic duct, and the ventral pancreatic duct [3]. The ventral pancreatic primordium arises on about the 32nd day from the base of the hepatic diverticulum and grows within the duodenal mesentery [2]. It has two lobes, right and left. The left lobe usually regresses, and only the right lobe with its ductal system remains [4]. The two pancreatic primordia meet on the 37th day with a displacement of the ventral primordium dorsally and to the right around the duodenal axis following the duodenal movements (described later), so that the common bile duct is found anterior to the dorsal pancreatic duct. The dorsal and ventral pancreatic ducts anastomose and the ventral primordium gives rise to part of the head and the uncinate process of the pancreas. The rest of the pancreas's head, tail, and body are derived from the dorsal primordium. It is obvious from the above that the pancreas, the distal common bile duct, and the duodenum are surgically, embryologically, and anatomically an “inseparable unit” [5].

The superior mesenteric artery (SMA) arises from the 9th, 10th, 11th, 12th, and, mainly, the 13th ventral segments of the primitive aorta at the level T1-4, and reaches its final position at L1 by the end of the second month of gestation, following the “caudal wandering” fashion described for the great abdominal vessels [6].

The herniation of the midgut into the embryonic coelom commences in the fourth week. The midgut begins to return, after its elongation and rotation around the mesenteric axis into the embryonic coelom, in the peritoneal cavity by the 10th week of gestation [7]. The duodenum does not exit the peritoneal cavity and is suspended by a mesentery. There it elongates, its antimesenteric part growing more rapidly than the mesenteric part, thus forming a loop containing within its mesentery the enlarging head of the pancreas. The growing liver pushes the stomach and subsequently the duodenum, so they rotate, and the left side of the duodenal mesentery faces anteriorly. The caudal tip of the duodenal loop reaches inferiorly and to the left of the SMA, which is “hugged” in variable degrees by the uncinate process of the pancreas [3,8]. This loop is displaced dorsally and to the right upon return of the midgut into the peritoneal cavity, followed by the pancreas, and they become fixed to the posterior abdominal wall [7]. Their mesentery is forced to fuse with the posterior parietal peritoneum covering the inferior vena cava and aorta, in a manner that the second, third, and fourth parts of the duodenum and pancreas are finally located retroperitoneally, and covered by peritoneum only anteriorly [9]. Laterally, the mesentery fuses with the anterior layer of the renal fascia [10]. The transverse mesocolon, during fixation of the colon, attaches to the second part and the corresponding area of the pancreas, leaving the site of attachment “bare” of the peritoneum [5].

Surgical anatomy items: how?

The liver is retracted upwards and the right colic flexure downwards. It is more convenient for the surgeon to stand on the left of the patient. From that side, the surgeon rolls towards his side the second part of the duodenum, so that the overlying parietal peritoneum is stretched. The incision is made about 3 cm from the rim of the duodenum, at a level about half the length of its second part, using a scalpel, and deep enough to reach the plane of the posterior surface of the duodenum. This plane is avascular and amenable to blunt dissection using the surgeon’s fingers. The incision extends perpendicularly to reach, cranially, the foramen of Winslow and caudally down to the flexure, where the third part of the duodenum begins. The duodenum is mobilized, the head of the pancreas following, and they are detached from the inferior vena cava and the aorta, with the superior mesenteric vessels being the limit for further mobilization [11]. The right gonadal vessels, the renal vessels, and the ureter are not visualized, as they are contained within the renal fascia, which should remain intact during the procedure. With the Kocher manoeuvre, only the first and proximal second part of the duodenum and the head of the pancreas can be mobilized. The attachment of the transverse mesocolon over the second part of the duodenum and correspondingly the pancreas requires that for the whole duodenum to be mobilized, and the cecum, ascending colon, and right colic flexure should be mobilized and reflected as well (Cattell manoeuvre) [12].

Underlying the duodenum and head of the pancreas, there is an avascular plane composed of loose connective tissue, which permits easy and bloodless detachment and mobilization [9]. It has been proved that it is a distinct fascia. The part underlying the duodenum and head of the pancreas is called the fusion fascia of Treitz, and it is continuous with the fusion fascia of Toldt, which underlies the body and tail of the pancreas [9]. This fascia and the pancreatic capsule are absent between the body of the pancreas and the SMA [13]. It can be divided, its ventral part following the duodenum and pancreas during mobilization, containing the major vascular arcades. Laterally, it fuses with the anterior leaf of the renal fascia, so on cross-section, the position of the pancreas and the duodenum is anterior to the kidneys, lying in the anterior pararenal space, because they are secondarily retroperitoneal organs [10,14].

Consequently, the point of incision of the peritoneum is crucial. If the incision is made more laterally, the renal fascia would be opened, and the correct plane of mobilization lost. Alternatively, if the incision is made too close to the duodenum, there is a danger that the vascular arcades could be injured, resulting in profound bleeding.

The uncinate process of the pancreas is derived from the ventral anlage [5]. Depending on its size, it "hugs" from behind in variable degrees, the SMA and vein. Besides that, the ligament of the uncinate process, if present, attaches the uncinate process firmly to these vessels, and, in cases of cancer, to the aorta, making total excision of the pancreas impossible.

What else then?

With the Kocher manoeuvre, (a) the mesoduodenum is restored, and the duodenum is rendered movable. The posterior surfaces of the duodenum and pancreas are visualized, and the hidden peripheral parts of the common bile duct are palpated [3]. The porta hepatis is exposed and easily dissected [15]. (b) This would not be feasible if the fascia of Treitz did not exist as a distinct anatomic structure. (c) The head of the pancreas is lifted and separated from the inferior vena cava and the aorta exposing them, but cannot be detached from the SMA [16]. (d) The foramen of Winslow is opened, the posterior aspect of the hepatoduodenal ligament can be clockwise rotated 180°, and the lesser sac is visualized [17]. The portal vein can be controlled in cases of haemorrhage [18]. (e) The SMA is the limit for further mobilization of the pancreatic head. Despite this fact, its supramesenteric segment is excellently visualized, and an aberrant right hepatic artery arising from the SMA can be identified and controlled. The artery can be easily dissected because of the sparse adipose tissue surrounding its retropancreatic segment. Its exposure by the Kocher manoeuvre makes it amenable to procedures such as embolectomy or aortomesenteric bypass [19].

Indeed, the Kocher manoeuvre remains the sole method for the mobilization of the duodenum and head of the pancreas. Despite its anatomical clarity, questions have arisen as to what extent it distorts other retroperitoneal anatomic structures, i.e., the veins of Retzius. These veins comprise one of the portocaval communication conduits in patients with portal hypertension. They are found, in most individuals, in the lower half of the second part and sometimes in the proximal third part of the duodenum, and open only when portal vein pressure is elevated [20]. That means that under normal circumstances, distortion is minimal. The kidneys, ureters, renal vessels, and gonadal vessels are contained within the perirenal compartment, which is enclosed in the renal fascia [10]. If the anterior layer is not mistakenly violated during the procedure, those structures cannot be harmed; in fact, they cannot even be visualized except in very thin individuals.

Identification of the plane where the fascia of Treitz is located is crucial for performing the Kocher manoeuvre. This fascia is made up of loose connective tissue and can be surgically divided. During fixation of the duodenum retroperitoneally, the two layers of peritoneum that meet are absorbed, and the primitive inner stratum gives rise to a fascia without histological features suggestive of peritoneum [13,14]. In foetuses, up to 20 weeks of gestation, the remnant of the mesoduodenum was found attached to the renal fascia. Later, during the 20th-30th week, the primitive fascia of Treitz begins to become evident histologically, and separated by loose connective tissue from the renal fascia [2]. It was long considered to be a fusion fascia, but Cho et al. suggest that its structure resembles the structure of migration fasciae and that this is the effect of the stress caused by movements of the duodenum as it rapidly grows and rotates [2].

Whatever the truth, its division leaves the pancreatic vascular arcades to the pancreatic side, which is covered with the ventral leaf. The aorta and inferior vena cava remain covered by loose connective, areolar, and adipose tissue. Major nerves and nerve plexuses, lymphatics, and lymph nodes remain on the side of the major vessels and are not mobilized during Kocher’s manoeuvre [21,22]. These structures comprise the mesopancreas, an entity that has been the subject of much debate lately, concerning its excision in cases of malignant tumours of the head of the pancreas.

The mesopancreas (or retroportal lamina or pancreatic plexus II) is considered the “mesentery” of the head and neck of the pancreas and is the tissue contained in the triangle between the posterior surface of the SMA and the portal vein, the anterior surface of the aorta between the origins of the SMA and celiac trunk (CT), limited on each side by the right semi-circumferences of the SMA and CT plexuses [23-25]. It represents a site of fusion of peritoneal layers and is continuous and inseparable through the structures contained in it, with the paraaortic region [26]. Similarly, the term mesopancreatoduodenum, an area that includes the left side of the SMA, has been proposed lately [27,28]. Mesopancreas is very frequently a site of invasion in pancreatic head tumours, and as a result of that, the reason for local recurrence [27]. Its complete excision is considered a prerequisite for a R_o_ excision of a pancreatic head tumour and is thought to improve survival rates, although there are not yet any randomized controlled trials to support it [29,30]. Extended lymphadenectomy has not been proven to offer any benefit in the survival of patients and only improves tumour staging and decision-making in multimodal treatment [26]. There are limitations in its "en bloc" complete excision, due to the nature of the structures contained in that area [31], and the fact there is no definite fascial/peritoneal anatomical plane that contains it, unlike the mesorectum, for example, whose boundaries are well-defined by fascial structures [24,31,32]. Apart from that, infiltration itself, inflammation, and fibrosis, which are common in cancer patients, distort normal tissue planes, a fact that per se can prevent proper excision on healthy margins [33]. Pancreatic cancer is spread through lymphovascular and perineural routes and surrounding fat by means of tumour budding and the formation of tumour deposits (endothelial-mesenchymal transition-related phenomena) [29]. The importance of complete and radical excision of mesopancreas for removing these neoplastic cell nests is clear, and there is evidence that dissection of the SMA on the subadventitial plane should be adopted in surgical practice [23]. It is a matter of R (resection) not N (nodal spread) factor of tumour resection [34]. Indeed, in most specimens with subsequent local recurrence, positive resection margins have been found to correspond to the area of the mesopancreas [35].

## Conclusions

And why is it so important after all?

The Kocher manoeuvre is just an example of how an embryologic event can be translated to anatomical knowledge, and how this knowledge can be applied to everyday practice. The event of secondary fixation of the duodenum and pancreas in the retroperitoneum implies that this event could be reversed, taking advantage of the fascial planes that are formed. The Kocher manoeuvre was not our subject, after all, its philosophy is.

The pancreas is one of the most difficult organs of the human body to handle. Impacted on the great abdominal vessels in the limited anterior pararenal compartment, with its head "hugged" by the duodenum and its tail "diving" into the splenic hilum, pancreas resection is a difficult procedure reserved for a few specialists. When it comes to malignancy, things get even more challenging: removal of a tumour that can spread early in its course in surrounding tissues and invade major vessels, overcoming fascial frontiers through perineural and lymphatic routes.

All these events are attributable to its embryology. Good knowledge of embryology can give answers translated to surgical anatomy, and delineation of surgical anatomy can give insights into unforeseen problems the surgeon comes up with. The Kocher manoeuvre is a simple procedure with an obscure or no meaning, for someone unaware of embryology and surgical anatomy. Surgeons should be trained on how to manipulate nature, not distort or destroy it, to achieve the best possible result. Besides, research progresses with a basic question: "why?", which becomes "how?", and after that "what else?".
